# Applications of Low Altitude Remote Sensing in Agriculture upon Farmers' Requests– A Case Study in Northeastern Ontario, Canada

**DOI:** 10.1371/journal.pone.0112894

**Published:** 2014-11-11

**Authors:** Chunhua Zhang, Dan Walters, John M. Kovacs

**Affiliations:** 1 Department of Geography and Geology, Algoma University, Sault Ste. Marie, Ontario, Canada; 2 Department of Geography, Nipissing University, North Bay, Ontario, Canada; University of Calgary, Canada

## Abstract

With the growth of the low altitude remote sensing (LARS) industry in recent years, their practical application in precision agriculture seems all the more possible. However, only a few scientists have reported using LARS to monitor crop conditions. Moreover, there have been concerns regarding the feasibility of such systems for producers given the issues related to the post-processing of images, technical expertise, and timely delivery of information. The purpose of this study is to showcase actual requests by farmers to monitor crop conditions in their fields using an unmanned aerial vehicle (UAV). Working in collaboration with farmers in northeastern Ontario, we use optical and near-infrared imagery to monitor fertilizer trials, conduct crop scouting and map field tile drainage. We demonstrate that LARS imagery has many practical applications. However, several obstacles remain, including the costs associated with both the LARS system and the image processing software, the extent of professional training required to operate the LARS and to process the imagery, and the influence from local weather conditions (e.g. clouds, wind) on image acquisition all need to be considered. Consequently, at present a feasible solution for producers might be the use of LARS service provided by private consultants or in collaboration with LARS scientific research teams.

## Introduction

With the primary objective of matching agricultural practice with crop and soil conditions, the use of Precision Agriculture (PA) technologies is considered one of the key directions in modern agriculture development. Some of the perceived benefits of PA include increasing crop yield and efficiency by lowering the costs associated with fertilizer, pesticides, herbicides, and fungicides. An additional socio-economic benefit of PA is reducing the transport of agriculture inputs on the air, soil and water. To date, considerable progress has been made in reducing the application of fertilizer, insecticides and fungicides using Variable Rate Technologies (VRT) and Global Positioning Systems (GPS). However, one main remaining challenge is the ability to obtain up-to-date crop/soil condition data (e.g., nutrient deficiency, water stress, pests, disease) for VRT. Historically, yield maps from yield monitors had been applied to create zonal maps for VRT machines [1]–[3]. However, these maps are normally obtained once a year and often the large variation observed make the reliability of the zonal maps limited [4]. Moreover, these types of yield maps are only available after the season, and many harvesters are still not equipped with yield monitors [5].

Alternatively, remotely sensed imagery obtained during the growing season could be utilized to extract crop condition information for management purposes in a timely fashion. In addition, yield maps derived from these data could be used as an alternative for yield maps from harvesters [5]. In particular, high spatial resolution satellite imagery can provide crop and soil condition information for management adjustment. For example, a variety of satellite data, including IKONOS, QuickBird, GeoEye-1 and WorldView-2, have been successfully applied in crop yield predictions [6]–[16]. However, image availability is highly restricted for these sensors due to weather condition and the satellites' poor temporal resolution. Moreover, the spatial resolution of these satellite images is limited with the highest resolution for commercial satellite data (WorldView-2 and GeoEye-1) at approximately 50 cm for the panchromatic band. Although quite good, this spatial resolution along with the limited spectral resolution of the panchromatic band might be not sufficient for examining within-field variations of crop condition and yield.

With finer spatial resolution and real-time monitoring capability [5], airborne multispectral [8], [17]–[19] and hyperspectral [11], [20] sensors had been applied to monitor crop conditions and yield. Aerial imagery has been shown to be as effective as high resolution satellite imagery in monitoring spatial variation of crop condition and yield. Furthermore, the rapid development of Low Altitude Remote Sensing Systems (LARS) over the past decade makes its application for PA possible. In 2000, Inoue et al. [21] collected crop images using a Charge-Coupled Device (CCD) camera on-board a blimp to measure biomass and Leaf Area Index (LAI) variation within rice and soybean fields. The results from their study showed that it might be plausible to apply LARS images in studying crop biological parameters. More recently, research scientists at the US Department of Agriculture have been conducting experiments using a fixed wing UAV to monitor various crop characteristics. Specifically, Hunt et al. [22] used a color digital camera on-board a radio controlled model aircraft to collect images of a corn field in order to examine the relationships between Normalized Green Ratio Difference Index (NGRDI), biomass and corn nitrogen status. Similarly, Hunt et al. [23] assessed the relationships between LAI and Green Normalized Difference Vegetation Index (GNDVI) for a wheat field. More recently, Hunt et al. [24] used a modified digital camera on-board a LARS to take high-resolution (i.e. 2.7 and 5.1 cm) color-infrared pictures of two winter wheat fields. They assessed the spectral information with ground collected biophysical data to demonstrate the scientific feasibility of applying LARS to monitor within-field crop variations. Most recently, Primicerio et al. [25] employed an ADC-lite camera on-board a UAV to acquire photos of a vineyard. They were able to convert digital numbers to reflectance and then calculated NDVI to display vineyard vigor. Peña et al. [26] and Torres-Sánchez et al. [27] both applied UAV images to map weeds in corn and sunflower fields, respectively.

While a number of sensors/cameras are available for LARS, optical (either metric or commercial scale) or infrared (metric or commercial with modified filter to record near infrared radiation [22], [23]) are the most commonly used for crop monitoring. Thermal infrared sensors have been shown to be useful for monitoring soil moisture or stress [28]–[30] and, most recently, hyperspectral sensors on board a UAV were used to examine leaf carotenoid content [31]. From the aforementioned studies, the number of crop types examined using LARS is still limited, mainly rice [21], [32], [33], soybean [21], wheat [24], [34], sunflower [27] and corn [22], [26], [35].

The studies to date demonstrate the scientific feasibility of LARS applications for monitoring crops. LARS appears capable of resolving the spatial resolution restrictions of satellite imagery. However, there are several key limitations apparent in such studies including the small spatial coverage and the image processing of the LARS data. For example, in Canada transportation regulations restrict the operating height of LARS, which means a large number of images need to be collected for each field. Depending on the percent of front- and side-lap of the images, a 30-acre field may require over 300 images. Moreover, because of the relative homogeneity of crops in the field, it is difficult to mosaic the images [24], [36]. Hunt et al. [24] reported that calculating NDVI or other vegetation indices from LARS image mosaics is challenging. For example, the same crop feature in several images could have different digital numbers due to changes in the incident angles and/or the atmospheric transmittance [24]. Consequently, most published LARS investigations focus on each image separately and not as an image mosaic [21]–[23], [25].

There appear to be mixed messages about the practical applications of LARS for PA. On one hand, the scientific research demonstrates the ability to quantify relationships between crop biomass [33] and water stress [29] with the digital numbers (or reflectance values) acquired from LARS imagery, which would suggest a very practical use for crop monitoring. On the other hand, the analyses are most often carried out on each image separately, which would not be practical for producers who may require hundreds, if not thousands of images to monitor their fields. In addition, there are very few examples of applied applications of LARS for crop monitoring in the literature and none so far based on actual requests from producers. Working in collaboration with cash crop producers, we use case studies based in northeastern Ontario, Canada, to explore and describe some applications of LARS mosaic imagery for crop monitoring: scouting, emergency response, and field trials.

## Study Area

This research takes place in the clay belt area within in the West Nipissing District of northeastern Ontario, Canada. The main cash crops grown in this region are soybean (*Glycine max*), wheat (*Triticum* spp.), barley (*Hordeum vulgare*), oat (*Avena sativa*) and canola (*Brassica napus*). The annual mean temperature is 3.8°C and the annual mean length of the growing season is 180 days, with a frost-free period of only 120 days. On average, the last spring frost is May 15, and the first fall frost is September 15. The annual precipitation is 1008 mm, in which 273 mm is snow. Agricultural production is influenced by acidic soil, which requires limestone to neutralize the soil pH. Even though the growing season is relatively short, fast growing crop varieties have shown to be successful for this region [37]. With such a short growing season it is necessary to monitor the field crop conditions in a timely fashion. Moreover, the large acreage and scattered distribution of the fields, typical of this region, makes personal visits and scouting of the fields a challenge [38], [39].

Producer requests were drawn from members of the North Eastern Ontario Soil and Crop Improvement Association (NEOSCIA). During the 2013 growing season and the spring of 2014, the research team was contacted several times by Steve Roberge of Ferme Roberge and Mitch DesChatelets of Leisure Farms. They requested that we analyze fertilizer field trials, field tile drainage conditions, crop damage from an armyworm [*Spodoptera frugiperda*] infestation, and lodging following a storm event. These farmers gave us permission to fly over their respective farms. Federal permission to fly the LARS over this region of Ontario was granted by Transport Canada (Special Flight Operations Certificate (SFOC) # 5812-15-33-2012-1).

## Equipment and Methods

**Figure 1:**
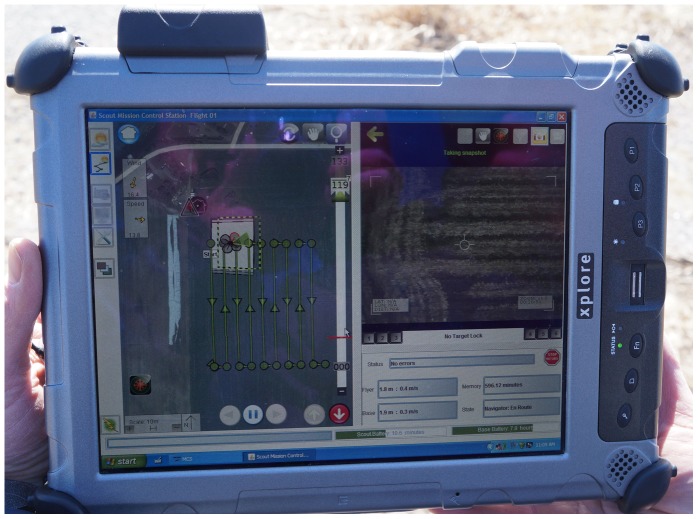
The touch-screen control station for the Aeryon Scout UAV.

**Figure 2:**
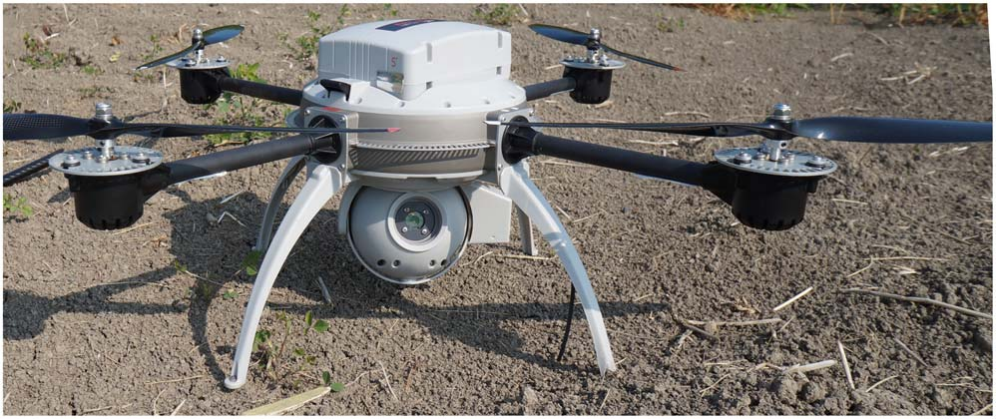
The Aeryon Scout quadrocopter.

For this study, the UAV system, developed by *Aeryon Labs Inc.*, Canada, consisted of a graphical, touch-screen control station (Figure 1), an aerial vehicle (Aeryon Scout) (Figure 2), and a radio repeater station to extend the control station's transmission range. This aerial vehicle is a commercially available quadrocopter UAV that can be equipped with both an optical and infrared camera. The Aeryon Scout has a maximum flight time of 25 minutes with a communication range of 3 km. The flyer has a maximum ground speed of approximately 50 km/hr and can remain stable in gusts exceeding 60 km/hr. Rechargeable lithium polymer batteries power the flyer and base station. The control station allows the user to create flight plans that can be reused at a later date. This aerial vehicle collects GPS/INS data for each photo, which are later used to orthorectify and create a mosaic image.

Optical images were captured using the Photo3S optical camera (*Aeryon Labs Inc.*, Canada) and near infrared images using an ADC-lite camera (*Tetracam*, United States) that affix to the Aeryon Scout. Both cameras use a Bayer filter to record the radiance from the ground targets. The Photo3S optical camera has three bands: blue, green and red. The images captured with this camera are stored in the flyer and then transferred to a hard drive after landing. The ADC-lite has three bands: near infrared, red, and green. The images captured with the ADC-lite are stored directly on a flash card located in the camera. The flight altitude was set at 120 meters, as per our SFOC. Consequently, the spatial resolution for the Photo3S optical and NIR images were 3.5 cm and 5 cm, respectively. Given the homogeneity of the crop fields the front overlap and side lap were 85% and 65% respectively. The high overlapping flight path helps to improve the efficacy of post-flight mosaic processing. The Aeryon Scout can cover close to 0.1 km^2^ (∼25 ac) per battery charge, flying at 12 km/hr. Moreover, although the rechargeable batteries can be replaced quite quickly, the Aeryon Scout needs to return to the landing/takeoff location during battery replacement.

Ground Control Points (GCP) were set up to help in the orthorectification and georeferencing of the final mosaic images. Each GCP was made of a 30 by 30 cm foam pad placed on the top of a wood stake at a height of 1.5 m. Based on the size of the field, 6 to 8 GCPs were dispersed throughout the field prior to each flight. Locations of the GCPs were recorded using a Trimble GeoXH GPS (*Trimble*, United States). Coordinates recorded from this GPS unit had a positional accuracy of less than 10 cm after real time differential analysis.

**Figure 3:**
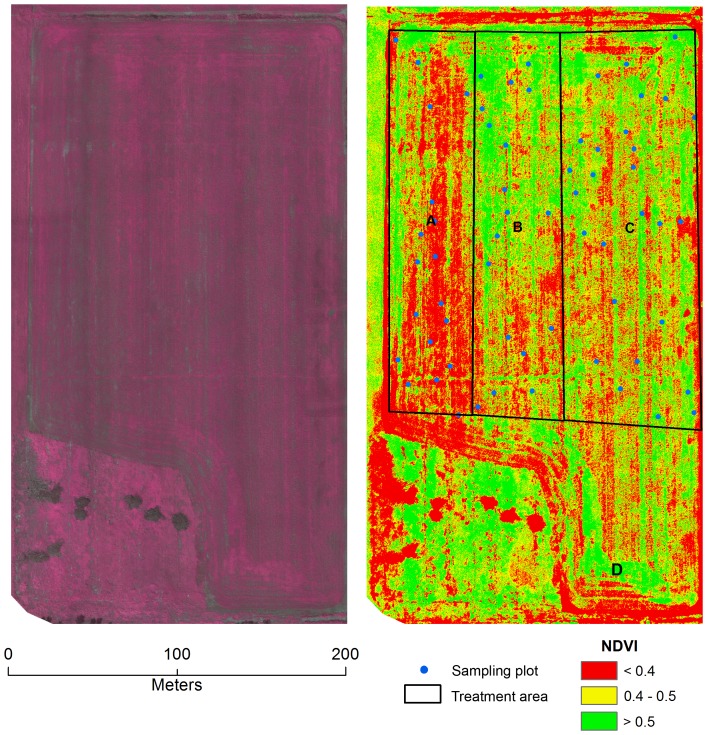
Mosaicked image map based on UAV images of a soybean field in Sturgeon Falls, ON, Canada (79°56′51″E, 46°20′14″N) taken on July 12, 2013. The image map on the left is a mosaicked infrared color composite image (NIR, red, green-no enhancement applied) and the image map on the right a mosaicked NDVI image. The A, B, and C represent treatment areas of organic only, organic and chemical fertilizer and chemical fertilizer only applications, respectively. D indicates a fertilizer application error. The final yields for the treatment areas A, B and C were calculated at 1.73, 2.27 and 2.97 tons/ha, respectively.

**Figure 4:**
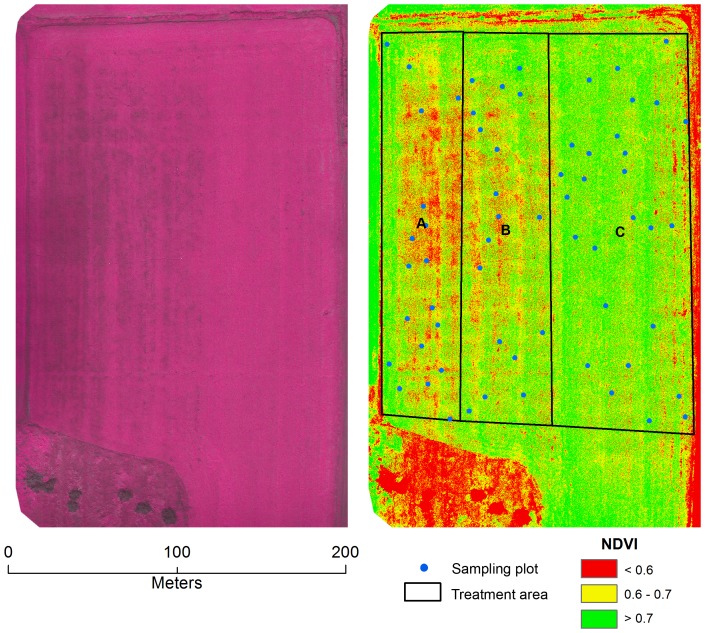
Mosaicked image map based on UAV images of a soybean field in Sturgeon Falls, ON, Canada (79°56′51″E, 46°20′14″N) taken on August 29^th^, 2013. The image map on the left is a mosaicked infrared color composite image (NIR, red, green-no enhancement applied) and the image map on the right a mosaicked NDVI image. The A, B, and C represent treatment areas of organic only, organic and chemical fertilizer and chemical fertilizer only applications, respectively. D indicates a fertilizer application error. The final yields for the treatment areas A, B and C were calculated at 1.73, 2.27 and 2.97 tons/ha, respectively.

For each mission, a team of three was required. One person operated the control unit for the planning and operation of the LARS, while the two others were responsible for flight observation (i.e. spotting aircraft or other potential hazards) and the distribution and collection of the GCPs. When using the ADC-lite infrared camera, a photo of a white Teflon calibration plate was taken upon takeoff for calibrating images taken. *Pixelwrench2* (*Tetracam, USA*) software was used to convert each raw image to a jpeg file and to calibrate the image. Field validation was done at the time the images were being taken. The optical and infrared imagery were then orthorectified and mosaicked using Pix4d Mapper (*Pix4D, Switzerland*) software. Pix4D was also used to generate NDVI images of fields. For the Leisure Farms soybean field a stratified random sample based on 1 m radius sample plots was used to statistically examine the differences in NDVI between the three fertilizer treatments. Specifically, a One-Way Analysis of Variance (ANOVA) was applied to mean values of 18 sample plots generated from treatment areas A and B and 27 sample plots generated from treatment area C (Figures 3&4).

## Results and Discussions

### The application of UAV imagery to assess fertilizer treatments

There have been several studies demonstrating the benefits of organic manure on soil quality and crop production. For example, adding compost has shown to increase crop production and improve soil fertility [40]–[43]. However, it is often necessary for producers to conduct their own trials in order to determine the economic feasibility of such products. In 2013 a local producer (Leisure Farms) conducted a trial test of an organic fertilizer on a soybean field to test the economic feasibility. He requested that the research team image of the field prior to an annual mid-season crop tour conducted by the members of the Nipissing District branch of the Ontario Soil and Crop Improvement Association (OSCIA). The three distinct fertilizer treatments were observed in the field (Figure 3). The producer had applied only organic fertilizer (9.37 L/ha or 1 gallon/acre) in section A of the field, whereas in section C a conventional chemical fertilizer (3–14–45, 371.25 kg/ha or 330 lb/acre) was applied. Section B (i.e. middle strip) was treated with a mix of organic (9.37 L/ha or 1 gallon/acre) and chemical fertilizer (185.53 kg/ha or 165 lb/acre). The research team flew his soybean field on a clear day (July 12, 2013) 42 days after seeding. The crop height was approximately 30 cm at the time image acquisition. The mosaicked image (Figure 3) shows a large contrast between the organic treatment and chemical fertilizer treatment. The section treated with only organic fertilizer had the weakest vegetation vigor and consequently appears much darker in the infrared image. The NDVI values are significantly lower than those of the chemical fertilizer treatment (P<0.001, Figure 3). However, there is no statistical difference between the strips of half organic/half chemical (B) and normal chemical fertilizer (C) application (P = 0.59). The observed variability within each treatment area could have been due to soil types, soil moisture content, or other factors. The large patch of high vigor (section D) in the southern section of the field was the result of operator forgetting to turn off the fertilizer spreader. The NDVI difference between treatments B and C were not detectible at the early stages of growth in July 12^th^ imagery. The images taken at later growth stages (August 29, 2013, 90 days after seeding, Figure 4) show greater variability among the three treatments. Significant differences (P<0.001) were observed between treatments A and C, and B and C. While the differences between treatment areas A and B were not statistically significant (P = 0.07), the P values is really close to the critical value of 0.05. Consequently, it is possible that a flight between these two dates would have provided better discrimination of the treatment areas.

### The application of UAV images in identifying area of lodging and insect infestation

**Figure 5:**
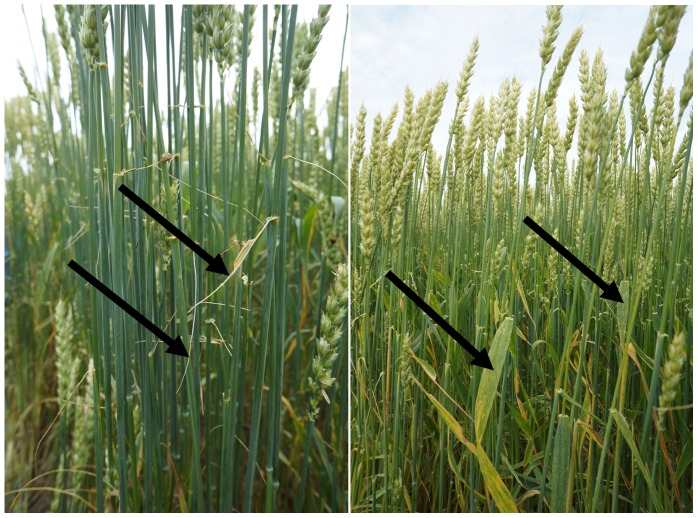
A comparison of the result of an armyworm attack. The arrows indicate the difference in the flag leaf of the infested (left) versus the healthy (right) wheat plants. Only the mid-rib of the flag leaves remains on the infested plants.

Fall armyworm is an agricultural pest more typical of tropical and subtropical regions. However, a cool, wet spring followed by warm, humid weather and heavy rainfall favor the propagation of fall armyworm in more temperate regions [44]. On average, one caterpillar needs 140 cm^2^ of leaf area to develop through 6 instars [44]. However, the 6^th^ instar itself requires 77.2% of that leaf area. Consequently, the producer in this study only recognized and reported the armyworm infestation at this stage growth. For many crops, including wheat, the fall armyworms tend to consume only the succulent parts of the leaves with the main midribs intact following the infestation (Figure 5). As a result the leaf area of the field or parts of the field drops significantly in a relatively short period of time. Given this type of damage it is believed that armyworm movement/impacts could be assessed using high resolution remotely sensed imagery. For areas infested within a wheat field, the reflectance in the NIR band should decrease whereas that of the red band should increase due to the loss of flag leaves and increased exposure of the soil surface and shadows. During the 2013 growing season the producer notified the research group that his wheat field was hit by the fall armyworm on July 31, 2013 when the wheat crop was at BBCH stage of 83. Consequently, a mission over the field was taken the following day under somewhat cloud covered conditions.

**Figure 6:**
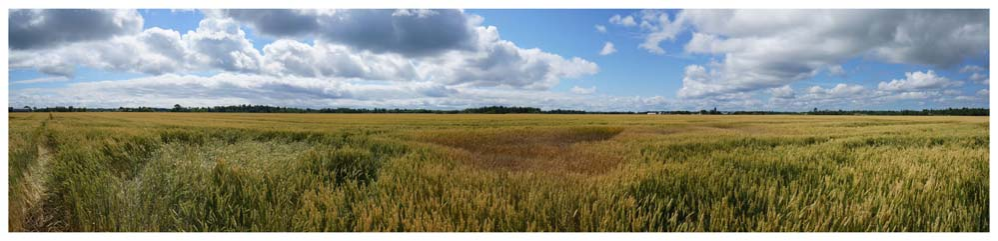
A picture showing lodging and crop stress in a wheat field on Roberge Farms. The photograph was taken between locations C and D in Figure 5, pointing south.

**Figure 7:**
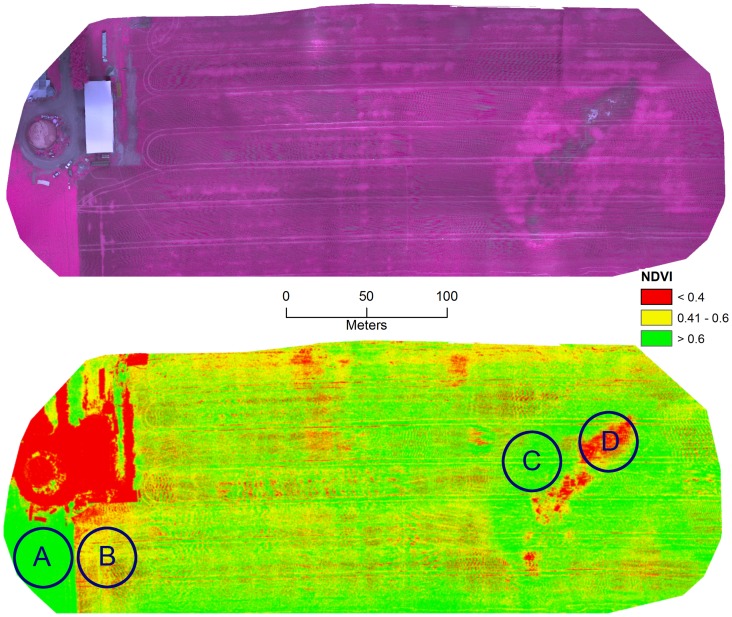
The top image is a mosaicked infrared color composite map (NIR, red, green-no enhancement) of a wheat field located in Verner, ON, Canada (80°5′50″E, 46°22′35″N) that was stricken by army worms and lodging taken on July 31, 2013. The bottom image is the corresponding NDVI derived map. The A indicates a healthy non-infested alfalfa field, the B indicates a section of the wheat crop hit by army worms, the C shows an area of lodging and D indicates a rock outcrop.

Lodging, or stem breakage, is a very common type of cereal crop damage which results from stormy weather events and the inadequate standing power of the crop during certain growth stages (i.e. heavy seed heads). Consequently, high nitrogen fertilization may cause plants to be more susceptible to lodging. For the Nipissing district a relatively strong storm event occurred on July 19, 2013 resulting in significant lodging within the same armyworm infested field (Figure 6). In the infrared image, the lodged areas appear as a bright red tone (Figure 7). The lodged wheat covers the bare soil and consequently there is stronger reflectance from wheat leaves and stalks in the IR band which results in the large contrast between the lodged and non-lodged areas.

In addition, it is also quite easy to identify stressed areas on and around the rock outcrop area (Figures 6 and 7). During the field trip the crops on the shallow soils were dead and are shown by a dark tone in the NIR images. The research team provided the producer with a mosaicked hard copy image and knowledge regarding how to interpret the data. The information was actually used to determine whether the producer should invest in equipment required to lift the lodged heads during harvesting.

### Using UAV images to identify a field tile drainage network

The main soil type for this area of northeastern Ontario is clay and the topography is nearly level with gentle slopes of 1–2%. The combination of clay and flat terrain has led to drainage problems for local producers. Consequently, field tile drainage systems are commonly installed to reduce the risk of crop loss from excess water and provide more uniform crop production amidst climate variability [45]. In addition, producers have higher flexibility in field operations (e.g., planting, drier harvest conditions, less soil compaction, and a wider choice of crops and crop varieties) [45], [46]. Good drainage can also reduce the frequency of pests and disease outbreak [45]. On tiled land, producers are able to obtain a modest return [47]. Once installed these systems need to be monitored and maintained and thus it is important for the farmer to know the exact location of their tiles. However, such information is not always available to farmers particularly when ownership of the field changes. In the Nipissing district it is common that the contractors only provide the producers with hand-drawn maps of the drainage system. In Ontario, the Ministry of Agriculture and Food normally maintains tile drainage information but access to their GIS database revealed very little coverage for this region of Ontario.

**Figure 8:**
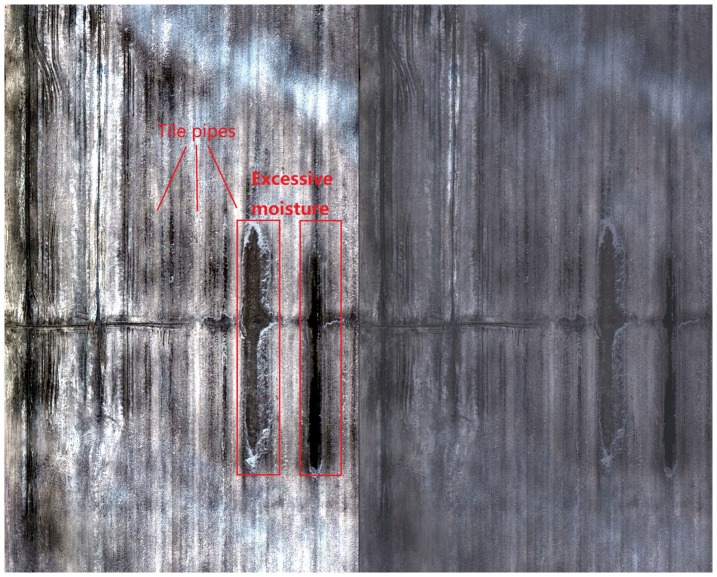
Mosaicked image maps, based on UAV optical images, of a bare field located in Sturgeon Falls, ON, Canada (79°56′51″W, 46°20′44″N). The image maps were used to locate the tile drainage pipes and to identify faulty drainage pipes. A linear enhancement applied to the optical image map (left) helps to better discriminate the tile drainage configuration in comparison to the non-enhanced optical image map (right).

The owner of Leisure Farms had two fields tiled in 2012 but was not able to obtain maps of the location of the tiles from the contractor. Consequently, he requested the research team identify the tile locations prior to seeding. On April 28, 2014 images were collected using the UAV system, processed and mosaicked. The mosaic image was then converted from a tiff file format to a KMZ and e-mailed to the producer for interpretation on Google Earth on the same day. In a follow up phone conversation with the producer we were able to identify locations of some of the tiles in the image which depicted a brighter tone with the expected linear feature. Areas well drained were drier and consequently look brighter (Figure 8). The interpretation is also validated by the fact that the field tile drainage network was located at the expected 50 feet (15 m) on centre interval. Further, we were also able to identify some drainage problems (i.e. excessive wetness) in the field possibly resulting from a poor grade during installation (Figure 8). Interpretation was possible in part to the bare soil present at this time. We were not able to identify the tile drainage system for another of his fields due to the presence of a residual straw cover. Based on the positive results, the producer suggested that we might receive more requests from other local producers to identify field tile locations and drainage problems.

### Weather conditions and UAV image acquisition

Weather conditions are critical for remote sensing acquisitions and unfortunately the growing seasons are typically the rainy seasons for many parts of the world. For example, the City of North Bay, located only 50 km east of the study area, experienced 30 rainy days in the 77 days from June 1^st^ to August 16^th^ of 2013. In addition, there were ten days with trace amounts of precipitation. Consequently, sky conditions can considerably hinder the availability of satellite imagery during the peak-growing season. In fact, our research group had requested a WorldView-2 image for the study area during the growing season for two consecutive years (2012–2013) without any success due to persistent cloudy conditions.

In comparison to satellite and high altitude aerial remote sensing, LARS has a higher degree of flexibility with regards to image acquisition. Although it is best to take imagery during a cloud free period, LARS images were successfully collected under full cloud cover. However, the impacts of varying solar radiation on the image should be considered for each task particularly when creating large mosaics based on hundreds of individual images.

### Factors impacting LARS adoption for PA

At present one of the major factors impeding the adoption of LARS is the cost. Currently it is expensive to own LARS equipment with the flyers alone costing between US$20,000 and US$70,000 [48]. The camera cost range from several hundred dollars for a standard commercial camera to upwards of US$7,000 for a metric infrared camera. The maintenance cost should also be considered. For example, the quadrocopter propellers can be damaged and rechargeable batteries have a limited number of cycles. Consequently, an additional US$5,000 should be budgeted for LARS maintenance. For this study an additional software expense was incurred to mosaic the imagery. Finally the costs of liability insurance must also be considered if the LARS is to be flown for commercial or research purposes in Canada and other jurisdictions. Fortunately, insurance was covered through our institution at no additional cost.

Another current limitation for the wide use of LARS for PA is the personnel required. A team of two individuals, one operator and one spotter, were required as part of the SFOC. However, it is recommended that at least three people be present. Specifically, a trained and qualified person needs to be responsible for the assembling, operation and disassembly of the UAV, a spotter is required and it is suggested that a third person set up the GCPs, measure the reference targets spectral responses, and act as a second spotter. In Canada a SFOC is also required for all commercial and research uses of UAV. The UAV team should also be able to mosaic images and georeferencing them shortly after image acquisition. In this investigation we determined that one of the team members needs to spend roughly one hour to download all of files associated with the UAV images, the GPS coordinates, and the spectral measurements. Moreover, another two to four hours, depending on the number of pictures collected, are required to orthorectify these images.

Besides the requirement of short image processing, certain skills in image interpretation or classification are also necessary for effective use of a LARS for PA. Most producers would require image interpretation training. The collection and processing of UAV images in a timely fashion is a key obstacle for their practical application. For our tile drainage example, we received the request from the producer on April 21, 2014. The fight was possible only due to ideal weather conditions and other logistical issues. The travel time from our institute to the study area is roughly 40 minutes. We went to the field on the morning of April 28, 2014 and finish four flights in just under two hours. We had to then travel back to our lab, download all the images and process on the same date. Two KMZ files were sent to the farmer that same day. In this tile drainage case, skills of image interpretation are very important [48]. The farmer was not able to visually identify tiles from the image mosaic until we sent him a KMZ file with our digitization of the tiles.

### Feedback from farmers on the application of UAV image in PA

During the process of image acquisition, we had many discussions with the farmers regarding the issues of LARS applications in agriculture. The owner of Leisure Farms was extremely satisfied with the mosaicked imagery we provided to him for the soybean fertilizer trials and tile drainage maps. Based on the results he decided not to bother including his soybean treatment in the annual crop tour of 2013. Moreover, he was impressed with the ability to identify the field tile drainage runs. He anticipated we would receive request for this service from other farmers in the area. Based on the extent of lodging in Roberge Farms wheat fields (approximately 13%) Steve Roberge decided to purchase a lift fork to harvest lodged wheat. According to these farmers, a fast response is the key for the application of UAV imagery in PA, especially for the requests of insect damage and other crop stress scenarios. Consequently it is critical to set up a routine procedure for image capture and processing. As a result, image processing (mosaic, georeferencing and interpretation) should be completed in one or two days with feedback directed to the farmer as quickly as possible. Considering the operating and processing costs of a LARS, it is more practical for a third-party to own the LARS and to provide the service.

## Conclusion

This paper examined the feasibility of applying UAV acquired images for monitoring crop conditions based on the actual requests from producers. The results suggest that it is plausible to obtain images and process them in a timely fashion for PA applications. However, due to current costs and operational logistics the application is still in its infancy stage. A fast adoption of UAV systems should occur as the costs of LARS decrease and more experienced personnel, possibly a service industry, are available to acquire and process these data in a timely fashion.
